# Serum inflammation-related proteins in a acute compartment syndrome rat model

**DOI:** 10.1038/s41598-024-83796-4

**Published:** 2025-01-02

**Authors:** Tao Wang, Jingcheng Cao, Zhiyong Hou, Qi Zhang

**Affiliations:** 1 Department of Lower Limb Trauma, Beijing Jishuitan Hospital, Guizhou Hospital, Baiyun District, Guiyang, Guizhou China; 2 Department of Orthopaedic Surgery, Third Hospital of Hebei Medical University, Shijiazhuang, Hebei P.R. China; 3 Orthopaedic Research Institute of Hebei Province, Shijiazhuang, Hebei P.R. China; 4 Department of Anesthesiology, Children’s hospital of Hebei Province, Shijiazhuang, Hebei province China; 5 Department of Anesthesiology, Hebei Children’s Hospital, No133 Jianhua South Street, Shijiazhuang, Hebei province China

**Keywords:** Acute compartment syndrome, Rat model, Proteomics, Inflammation, Immunology, Biomarkers

## Abstract

We aim to explore variations of serum inflammation-related proteins in an acute compartment syndrome (ACS) rat model. We collected serum from 25 healthy Sprague-Dawley rats (control group, CG) and 50 rats with tibial fractures, including 25 rats with ACS (ACS group, AG), and 25 rats without ACS (fracture group, FG). Ten samples per group were randomly chosen for proximity extension assay analysis of 92 inflammation-related proteins, and all samples were verified by enzyme-linked immunosorbent assays. Receiver-operating characteristic curve analysis was used to identify the diagnostic ability and cut-off values. Our findings showed that the levels of Il6 and Prdx5 in the FG and Il6, Prdx5, Dctn2, and Plin1 in the AG, were significantly higher than those in the CG. Notably, compared with the FG, high expression of Prdx5, Dctn2, and Plin1 was observed in the AG. Additionally, we identified 58.8764, 14.023, and 31.8730 pg/ml as the cut-off values of Prdx5, Dctn2, and Plin1 to predict ACS in rats. Similarly, the cut-off values of Il6, Prdx5, Dctn2, and Plin1 to predict ACS in healthy rats were 10.6783, 766.5879, 12.5627, and 14.3623 pg/ml, respectively. Furthermore, the combination of these proteins had the highest diagnostic accuracy. We identified Prdx5, Dctn2, and Plin1 as potential biomarkers of ACS compared with fracture in rats and revealed that combination of Il6, Prdx5, Dctn2, and Plin1 had the highest diagnostic accuracy to predict ACS compared with the healthy condition. Furthermore, the cut-off values for these biomarkers were determined, providing a new method to rapidly assess the risk of ACS and manage early targeted interventions.

## Introduction

Acute compartment syndrome (ACS), a serious orthopedic emergency, affects 1–30.4% of patients with lower extremity fractures according to published studies^1–3^. A rapid increase in pressure within a closed compartment after trauma causes bleeding, tissue edema, and reduced limb perfusion, resulting in tissue ischemia. A delay in diagnosis or treatment can cause adverse outcomes, such as sensory deficits, paralysis, infection, or muscle necrosis^4^, which presents challenges for orthopedic surgeons. Clinically, diagnosis of ACS is commonly based on clinical symptoms (pain, pain with passive stretching, and swelling) and clinician experience rather than the gold standard tests or diagnostic biomarkers. Thus, identification of biomarkers to predict ACS by application of high-throughput technologies is urgently needed.

Regarding the animal ACS model, tourniquets and infusion techniques have been widely used in published papers^5,6^. However, these methods have obvious shortcomings. First, similar to the human ACS model induced by a tourniquet, the animal models may not accurately simulate a perfusion impairment such as that occurring in ACS. Second, infusion techniques are invasive methods and may result in increased extracellular volume instead of tissue edema. Third, these two methods are unlikely to clinically simulate the injury mechanism of ACS caused by injuries from an external source. Daly^7^ used a combination of crush injury and infusion techniques to mimic ACS in a rabbit model, which seems to be effective and needs to be verified and validated. We established a combination of crush injury and tourniquets to produce an ACS rat model, which can firmly reach the diagnostic gold standard of ΔP < 30 mmHg (ΔP = diastolic arterial pressure-intra − compartmental pressure)^8^.

Inflammation has been considered a major driving factor in the development of ACS in previous research^9^. Proteomics techniques have tremendously improved during the last decade and have been extensively used to screen for biomarkers of diseases^10^ because of the excellent reproducibility and stability of Olink technology. Additionally, this technology not only offers various assay panels targeted toward different diseases but also requires small sample volumes, which is crucial because of the limited number of clinical samples^11^. The development of proteomics technologies will advance our understanding of diseases, help identify biomarkers with clinical values, and ultimately contribute to human health. To our knowledge, this is the first study to explore the predictive proteins for ACS using Olink Proteomics in a novel ACS rat model.

## Materials and methods

### Animals

All experiments were approved by the animal care and the ethics committee of our hospital (S2020-024-1) and complied with the National Institutes of Health Guidelines for the Care and Use of Laboratory Animals. Our experimental procedures on rats are consistent with the ARRIVE guidelines. Rats were maintained on standard chow and housed under a 12-hour light/12-hour dark cycle and controlled temperature (22–24 °C) and humidity (50–65%). All experiments were performed at similar times of the day to prevent any circadian rhythm effects on the rats. Male 12-week-old Sprague-Dawley rats were purchased from the Beijing Vital River Laboratory Animal Technology Co. (Beijing, China).

## ACS rats model

**Fig. 1:**
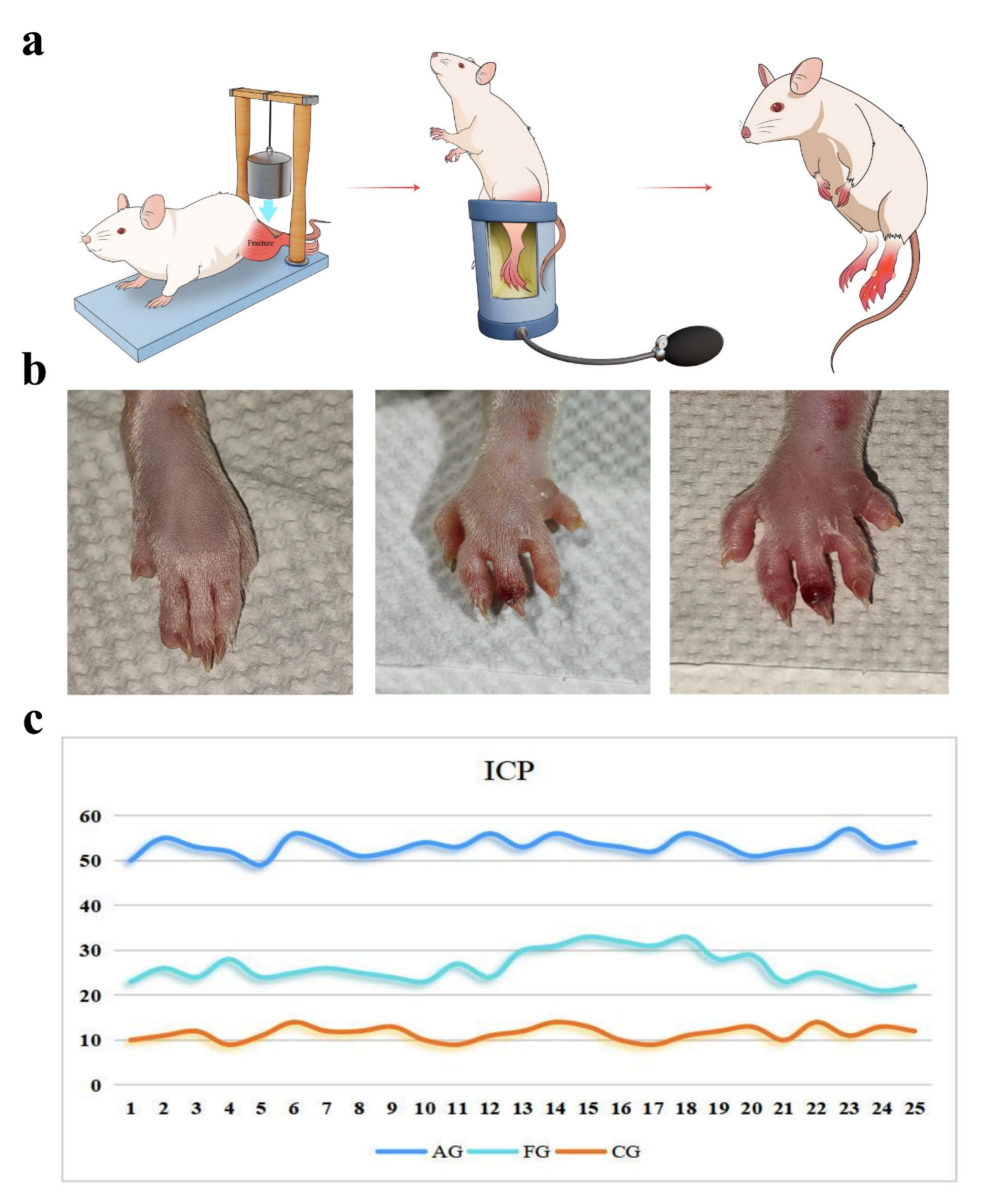
Chracteristics of ACS rats. (**a**) procedure of ACS rats; (**b**) swelling of rat paws; c.Intracompartmental pressure of the affected limb in three groups of rats. *AG: acute compartment syndrome group; FG: fracture group; CG: control group.

All 12-week-old male rats were randomly assigned to experimental groups. Blunt trauma was induced using a custom-made device. Different weights falling freely from a height of 40 cm onto the lower leg of the rat were used to cause various degrees of trauma, as described by Altay^11^. One hours after blunt trauma induction, a novel compression device, consisting of a pressure gauge cuff (DRO-SX, Constant Hui Medical Instruments LTD.) embedded in a rigid plastic tube, was used to compress the lower leg through the cuff, applying varying pressures for 6 h. The sample size for the experiment was determined according to a previous preliminary experiment (Fig. 1a).

We used Panlab NIBP System (Harvard Apparatus) to measure blood pressure in the tail of rats when the tail vessels were dilated and the pulse wave was stable. Tail arterial blood pressure was measured and averaged at least three times for each rat, with an interval of more than 5 min between each measurement. Then, we used an automatic intra-compartmental pressure measuring instrument (mmHg, CYY-1, Liyang Wanda Electronics Co. LTD.) to measure intra-compartmental pressure. Then, we can compute the ΔP (ΔP = diastolic arterial pressure − intra-compartmental pressure) using arterial blood pressure and intra-compartmental pressure.

After anesthetizing rats with isoflurane, we opened the rats’ chest cavity and used a 5 ml syringe to collect blood from the heart apex from 25 healthy rats and 50 rats with tibial fracture (25 rats without ACS and 25 rats with ACS), which were divided into a control group (CG), fracture group (FG), and ACS group (AG), respectively. After collecting the blood samples from the rats, we euthanized them by cervical dislocation. We obtained the serum by centrifugation at 3000 rpm for 10 min and stored in a − 80 °C freezer for later use. Finally, 10 samples from each group were analyzed using a proximity extension assay of 92 inflammation-related proteins, and another 15 samples were verified by enzyme-linked immunosorbent assays (ELISAs). The diagnostic criterion for ACS is as follows: ΔP < 30 mmHg (ΔP = diastolic arterial pressure − intra-compartmental pressure). The inclusion criteria for ACS rats were as follows: (1) rats with closed tibial fracture; (2) ΔP < 30 mmHg.

## Analysis of inflammation-related proteins

Protein levels were measured using the Olink^®^ target 92 inflammatory panel (Olink Proteomics, LC-Bio Technology Co., Ltd. Hangzhou, China) in accordance with the manufacturer’s instructions. In short, the target proteins in the samples were exposed to the binding of antibody probe pairs that had been labeled with oligonucleotides. When two antibodies linked tightly to one another, the DNA oligonucleotides expanded and hybridized via DNA polymerization to produce a reporter sequence for a polymerase chain reaction. Protein abundance was represented by normalized protein expression (NPX), an arbitrary unit on a log2 scale. A high NPX value indicates a high level of protein. Nevertheless, it is not possible to compare NPX values across distinct proteins.

To determine which proteins were differentially expressed (DEPs) between groups, the R package “OlinkAnalyze” was utilized. Principal component analysis was carried out using the R package’s “princomp” function, which identifies the most significant features of data variability. Heat maps, volcano plots, and enrichment analyses for Kyoto Encyclopedia of Genes and Genomes (KEGG)^12–14^ and Gene Ontology (GO) were produced using the ggplot2 program. Additionally, Spearman’s correlation was used to analyze the correlation between the expression of two proteins. Using Cytoscape (version 3.9.1), a protein-protein interaction (PPI) network of DEPs was created and displayed.

## ELISA validation

We used Peroxiredoxin 5 (Prdx5), Interleukin-6 (Il-6), Dynactin-2 (Dctn2), and Perilipin 1 (Plin1) ELISA kits (MB-0074 A, Jiangsu Meibiao Biological Technology, China) to be further verified.

### Statistical analysis

The study was performed using R software and SPSS (version 25.0 SPSS Inc., Chicago, IL, USA). A p-value < 0.05 is supposed to be statistically significant. When data conforms to continuous variables, the Student’s t-test was applied to assess and present the mean ± standard deviation. The Mann-Whitney U test was employed to compare groups statistically when the data were non-normal. The count data was analyzed using the Chi-square test. Additionally, the ROCR software was used to build receiver operating characteristic (ROC) curves in order to determine the best predictors of ACS.

## Results

**Fig. 2:**
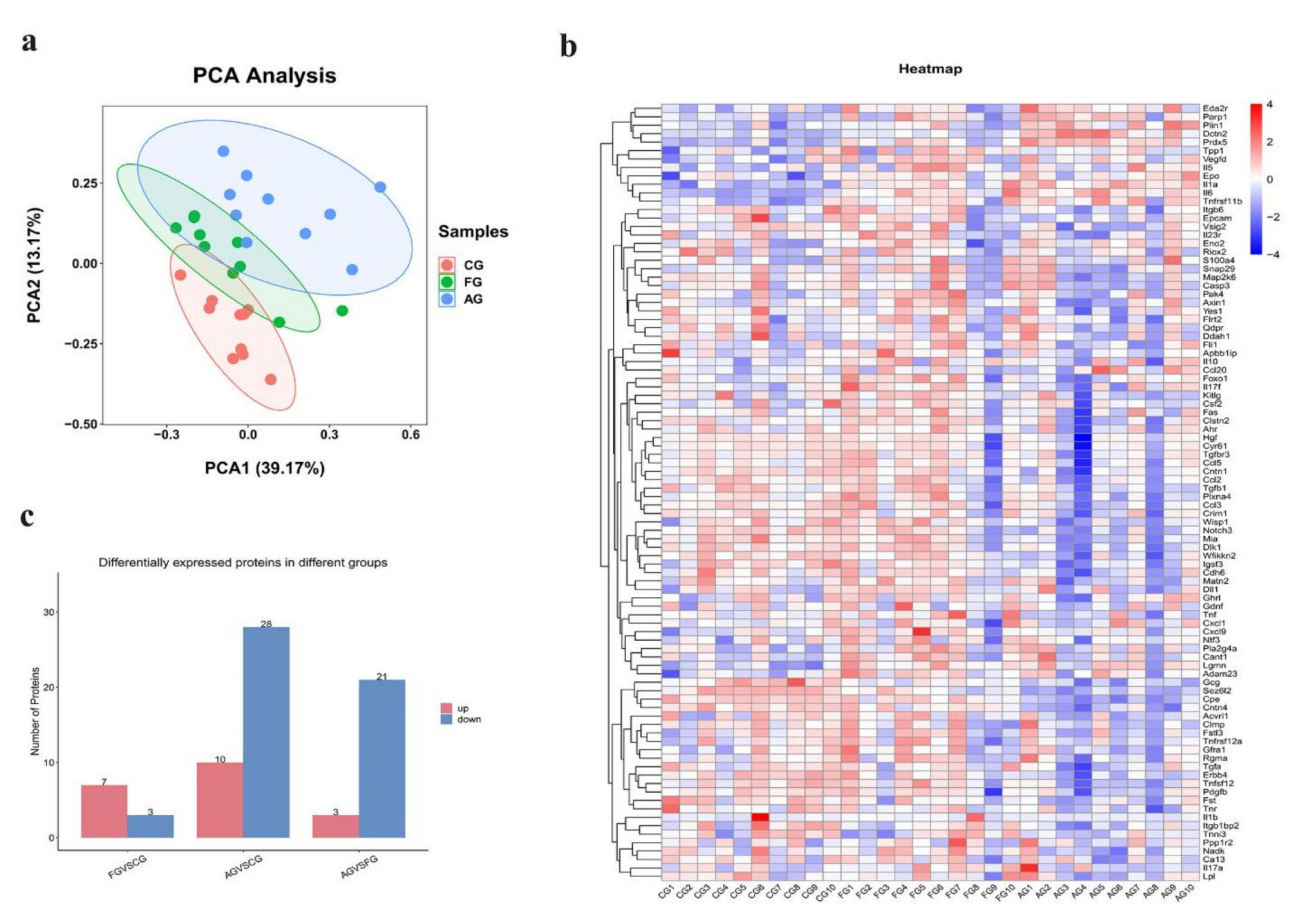
Comparison of the serum inflammation-related proteins among three groups. (**a**) Principal component analysis (PCA) showed two dimensions among 3 groups differentiated by color. Each point represents a single rat, with rats of similar protein expression profiles positioned next to each other; (**b**) Heatmap of protein expression among three groups. The X-axis shows the sample group and the Y-axis shows the protein name. Different colors represent different levels of protein expression, with blue to red representing low to high levels of expression; (**c**) Compare the number of differential proteins among three groups. (CG: control group; AG: acute compartment syndrome group; FG: fracture group)

Principal component analysis was used to observe similarities among the three groups. As for the variance, the first component was 39.17% and the second component was 13.17% (Fig. 2a). A heatmap was used to show protein expression among the three groups (Fig. 2b). Additionally, the numbers of up-regulated and down-regulated differential proteins were determined in pairwise comparisons (Fig. 2c).

### FG vs. CG

**Fig. 3:**
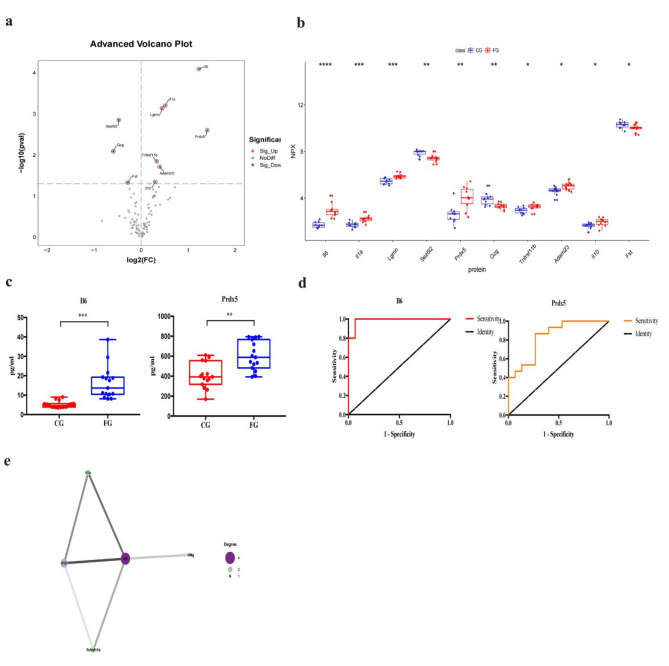
Comparison of the serum inflammation-related proteins between tibial fracture group (FG) and control group (CG). (**a**) Volcano plots showed significant differences of cytokines between FG and CG. Proteins highly expressed in FG were labeled in red. Proteins highly expressed in CG were labeled in blue. Differences between FG and CG were expressed as Log2 (fold change) of plasma on the x-axis and the (-Log10) p value on the y-axis; (**b**) Box plot representing differentially expressed proteins between FG and CG; (**c**)The levels of Il6, and Prdx5 between FG and CG were shown in box plot by ELISA; (**d**) Receiver operating characteristic curve analysis of Il6, and Prdx5 between FG and CG; (**e**) Correlation in protein abundance PPI using String. (CG: control group; FG: fracture group; * <0.05;**<0.01: ***<0.001)

As shown in Tables 1 and 10 differentially expressed inflammation-related proteins were found in two groups, including three down-regulated DEPs and seven up-regulated DEPs (Il6, Il1a, Lgmn, Prdx5, Tnfrsf11b, Adam23, and Il10) (Figs. 2c and 3a–b). ELISAs showed that the expression levels of Il6 and Prdx5 were markedly higher in the FG than in the CG (all *p* < 0.001, Fig. 3c). ROC curve analysis was used to calculate the area under the curve (AUC) of Prdx5 [*p* = 0.002, AUC = 0.836, 95% confidence interval (CI) (0.693 − 0.979)] and Il6 [*p <* 0.0001, AUC = 0.987, 95% CI (0.955 − 1.000)] (Fig. 3d; Table 2). We identified their cut-off values as 435.9519 pg/ml and 8.1486 pg/ml, respectively (Table 2). We also determined that Prdx5 + Il6 [*p* < 0.0001, AUC = 0.996, 95% CI (0.981, 1.000)] had the highest diagnostic accuracy (Fig. 3d; Table 2). Additionally, we investigated the interactions of up-regulated DEPs using correlation analysis and the PPI network, which showed that Il6 may play an important role in the FG (Fig. 3e).

**Table 1:** Protein expression analysis among three groups.

Protein	Groups			*P* value		
	CG (*n* = 10)	AG (*n* = 10)	FG (*n* = 10)	FG vs. CG	AG vs. CG	AG vs. FG
Acvrl1	3.54	3.05	3.60	0.83	0.09	0.04
Adam23	4.63	4.86	5.02	0.02	0.18	0.36
Ahr	1.00	0.86	1.06	0.47	0.26	0.12
Apbb1ip	1.25	1.13	1.29	0.77	0.34	0.17
Axin1	3.50	3.34	3.79	0.10	0.46	0.07
Ca13	3.66	3.22	3.43	0.44	0.15	0.52
Cant1	2.74	2.85	3.00	0.10	0.47	0.44
Casp3	7.46	6.58	7.41	0.94	0.15	0.24
Ccl2	12.60	12.38	12.55	0.51	0.01	0.11
Ccl20	1.90	2.16	1.99	0.55	0.15	0.39
Ccl3	8.04	7.00	7.85	0.61	0.00	0.07
Ccl5	10.71	10.03	10.74	0.94	0.11	0.16
Cdh6	3.60	3.14	3.54	0.65	0.01	0.02
Clmp	7.90	7.69	7.79	0.43	0.11	0.56
Clstn2	2.59	2.32	2.66	0.51	0.07	0.04
Cntn1	9.34	9.07	9.28	0.28	0.01	0.03
Cntn4	3.18	2.44	3.02	0.09	0.00	0.00
Cpe	6.46	5.23	6.21	0.07	0.00	0.00
Crim1	9.76	8.78	9.42	0.34	0.04	0.22
Csf2	1.10	1.04	1.18	0.52	0.60	0.21
Cxcl1	9.54	9.61	9.55	0.98	0.84	0.91
Cxcl9	5.53	5.58	6.10	0.12	0.82	0.14
Cyr61	9.05	8.55	8.86	0.37	0.06	0.32
Dctn2	2.42	4.13	2.71	0.10	0.00	0.00
Ddah1	3.12	2.83	3.03	0.79	0.46	0.54
Dlk1	8.50	8.15	8.46	0.70	0.00	0.02
Dll1	5.95	5.92	6.11	0.16	0.84	0.17
Eda2r	8.38	9.02	8.68	0.17	0.00	0.15
Eno2	3.71	3.88	3.72	0.90	0.19	0.29
Epcam	2.87	2.56	2.82	0.84	0.21	0.23
Epo	4.55	5.35	5.26	0.06	0.06	0.75
Erbb4	5.04	4.42	4.82	0.11	0.00	0.01
Fas	1.41	1.27	1.46	0.61	0.32	0.23
Fli1	1.03	0.98	1.28	0.07	0.73	0.06
Flrt2	5.43	5.27	5.65	0.28	0.41	0.08
Foxo1	1.50	1.40	1.66	0.19	0.31	0.06
Fst	10.29	9.81	10.00	0.05	0.00	0.18
Fstl3	6.79	6.67	6.82	0.81	0.27	0.23
Gcg	3.92	2.73	3.32	0.01	0.00	0.00
Gdnf	4.13	4.01	4.20	0.83	0.66	0.56
Gfra1	5.13	4.92	5.25	0.58	0.29	0.14
Ghrl	5.12	5.05	5.29	0.27	0.70	0.17
Hgf	11.54	10.93	11.26	0.22	0.06	0.36
Igsf3	6.34	5.92	6.25	0.38	0.00	0.01
Il10	1.64	1.70	1.94	0.05	0.76	0.25
Il17a	3.20	3.31	3.81	0.11	0.83	0.36
Il17f	1.08	1.04	1.26	0.05	0.75	0.09
Il1a	1.70	2.37	2.21	0.00	0.00	0.20
Il1b	2.26	1.48	2.05	0.82	0.36	0.22
Il23r	2.08	1.90	2.08	0.96	0.20	0.24
Il5	1.61	1.90	1.93	0.08	0.04	0.89
Il6	1.70	3.05	2.93	0.00	0.00	0.61
Itgb1bp2	3.60	3.03	3.35	0.25	0.02	0.09
Itgb6	2.92	2.56	2.82	0.53	0.02	0.12
Kitlg	1.73	1.48	1.81	0.47	0.10	0.02
Lgmn	5.45	5.81	5.89	0.00	0.01	0.50
Lpl	6.35	5.92	6.25	0.77	0.24	0.40
Map2k6	4.67	4.01	4.48	0.52	0.06	0.23
Matn2	5.53	5.08	5.71	0.42	0.08	0.03
Mia	6.68	6.22	6.55	0.26	0.00	0.02
Nadk	7.81	7.28	7.98	0.51	0.07	0.02
Notch3	5.65	5.24	5.52	0.15	0.00	0.02
Ntf3	3.74	3.75	3.87	0.44	0.95	0.46
Pak4	2.08	2.07	2.29	0.23	0.91	0.23
Parp1	1.14	1.48	1.30	0.19	0.00	0.18
Pdgfb	9.83	8.72	9.36	0.31	0.01	0.24
Pla2g4a	7.06	7.25	7.43	0.06	0.33	0.41
Plin1	1.82	2.47	1.92	0.56	0.00	0.01
Plxna4	5.16	4.33	5.29	0.75	0.04	0.07
Ppp1r2	2.52	2.63	2.64	0.65	0.63	0.99
Prdx5	2.61	5.03	4.02	0.00	0.00	0.02
Qdpr	9.08	8.73	8.90	0.59	0.36	0.65
Rgma	4.24	4.13	4.30	0.62	0.37	0.27
Riox2	2.44	2.28	2.34	0.65	0.42	0.74
S100a4	8.76	8.91	8.87	0.55	0.42	0.84
Sez6l2	7.89	6.91	7.41	0.00	0.00	0.00
Snap29	7.76	7.67	8.16	0.16	0.76	0.16
Tgfa	3.51	2.92	3.36	0.42	0.02	0.11
Tgfb1	5.95	4.86	5.82	0.76	0.00	0.03
Tgfbr3	11.81	11.66	11.79	0.80	0.08	0.18
Tnf	2.03	1.84	2.14	0.35	0.05	0.03
Tnfrsf11b	2.93	3.36	3.26	0.01	0.00	0.44
Tnfrsf12a	7.10	6.99	7.12	0.94	0.30	0.42
Tnfsf12	5.80	4.99	5.65	0.49	0.00	0.02
Tnni3	12.44	10.87	11.60	0.40	0.04	0.39
Tnr	2.81	2.53	2.69	0.18	0.02	0.11
Tpp1	2.66	2.70	2.91	0.11	0.77	0.09
Vegfd	4.12	4.35	4.38	0.10	0.12	0.80
Vsig2	1.73	1.36	1.63	0.59	0.03	0.20
Wfikkn2	10.14	9.73	10.04	0.14	0.00	0.01
Wisp1	5.60	5.17	5.63	0.84	0.01	0.00
Yes1	2.97	2.69	2.81	0.42	0.26	0.62

*CG = control group; AG = acute compartment syndrome group; FG = fracture group.

**Table 2:** ROC curve analysis and cut-off value between two paired groups.

Variables	Area	*P*-value	95%CI		Cut-off value
			Lower limit	Upper limit	
**AG vs. FG**					
Prdx5	1.000	<0.0001	1.000	1.000	858.8764 pg/ml
Plin1	0.942	<0.0001	0.855	1.000	31.8730 pg/ml
Dctn2	1.000	<0.0001	1.000	1.000	14.023 pg/ml
Prdx5 + Plin1	1.000	<0.0001	1.000	1.000	NA
Prdx5 + Dctn2	1.000	<0.0001	1.000	1.000	NA
Dctn2 + Plin1	1.000	<0.0001	1.000	1.000	NA
Prdx5 + Dctn2 + Plin1	1.000	<0.0001	1.000	1.000	NA
**AG vs. CG**					
Il6	1.000	<0.0001	1.000	1.000	10.6783 pg/ml
Plin1	0.996	<0.0001	0.981	1.000	14.3623 pg/ml
Dctn2	1.000	<0.0001	1.000	1.000	12.5627 pg/ml
Prdx5	1.000	<0.0001	1.000	1.000	766.5879 pg/ml
Il6 + Plin1	1.000	<0.0001	1.000	1.000	NA
Il6 + Dctn2	1.000	<0.0001	1.000	1.000	NA
Il6 + Prdx5	1.000	<0.0001	1.000	1.000	NA
Plin1 + Dctn2	1.000	<0.0001	1.000	1.000	NA
Plin1 + Prdx5	1.000	<0.0001	1.000	1.000	NA
Dctn2 + Prdx5	1.000	<0.0001	1.000	1.000	NA
Il6 + Plin1 + Dctn2	1.000	<0.0001	1.000	1.000	NA
Il6 + Plin1 + Prdx5	1.000	<0.0001	1.000	1.000	NA
Il6 + Dctn2 + Prdx5	1.000	<0.0001	1.000	1.000	NA
Plin1 + Dctn2 + Prdx5	1.000	<0.0001	1.000	1.000	NA
Il6 + Plin1 + Dctn2 + Prdx5	1.000	<0.0001	1.000	1.000	NA
**FG vs. CG**					
Il6	0.987	<0.0001	0.955	1.000	8.1486 pg/ml
Prdx5	0.836	0.002	0.693	0.979	435.9519 pg/ml
Il6 + Prdx5	0.996	<0.0001	0.981	1.000	NA

*CG = control group; AG = acute compartment syndrome group; FG = fracture group.

**Fig. 4:**
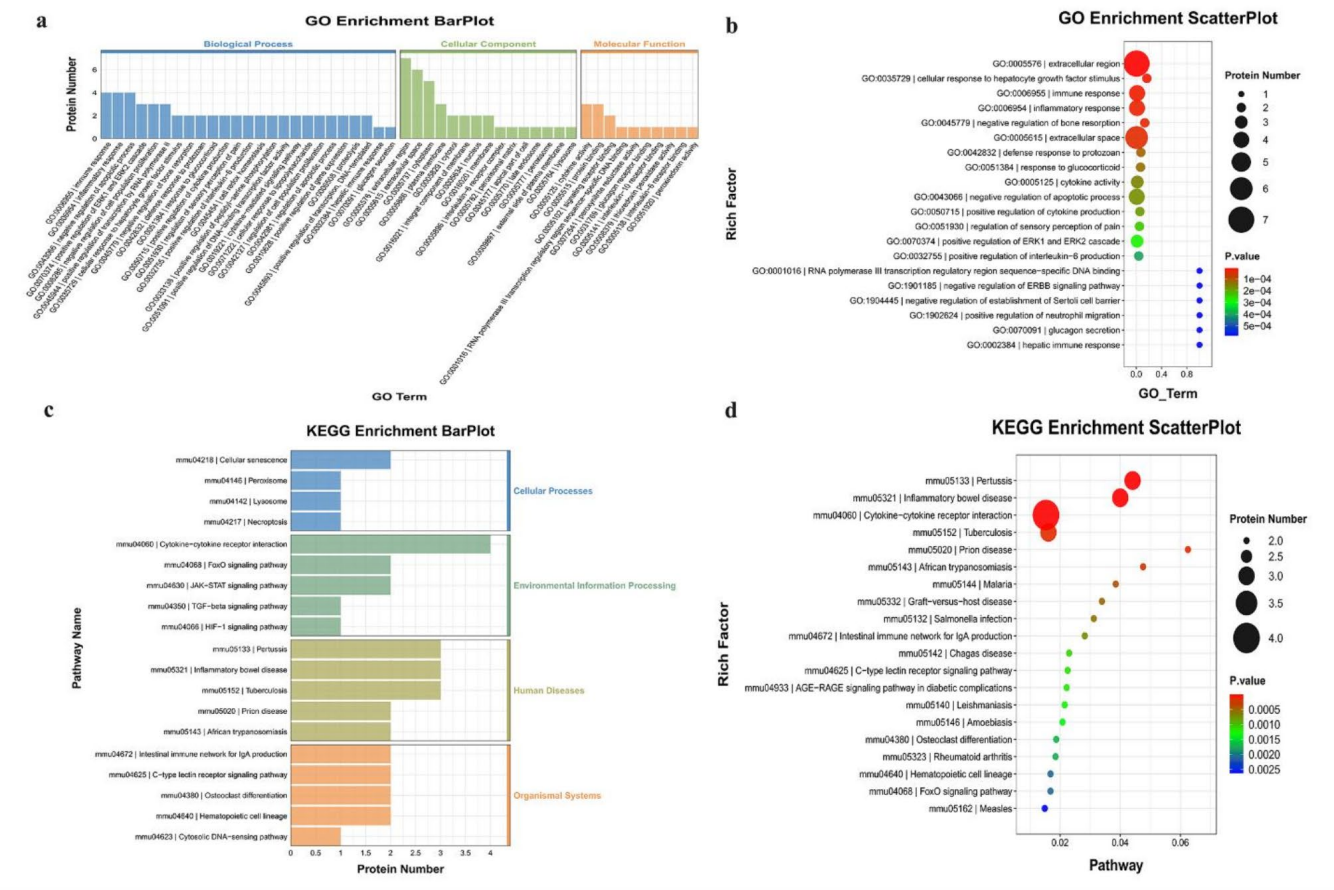
Gene Ontology (GO) terms and Kyoto Encyclopedia of Genes and Genomes (KEGG) of the significant inflammation proteins between FG and CG. (**a**&**b**) GO terms of the significant inflammation proteins between FG and CG; (**c**&**d**) KEEG enrichment analysis of the significant inflammation proteins between FG and CG. (CG: control group; FG: fracture group)

Additionally, GO and KEGG enrichment analyses were performed to investigate the potential functions of the up-regulated DEPs in the FG. According to GO enrichment analysis, the immune response, cellular response to hepatocyte growth factor stimulus, and inflammatory response were enriched (Fig. 4a–b). Additionally, KEGG enrichment analysis indicated that cytokine-cytokine receptor interaction, the FoxO signaling pathway, and the JAK-STAT signaling pathway were enriched (Fig. 4c–d).

### AC vs. CG

**Fig. 5:**
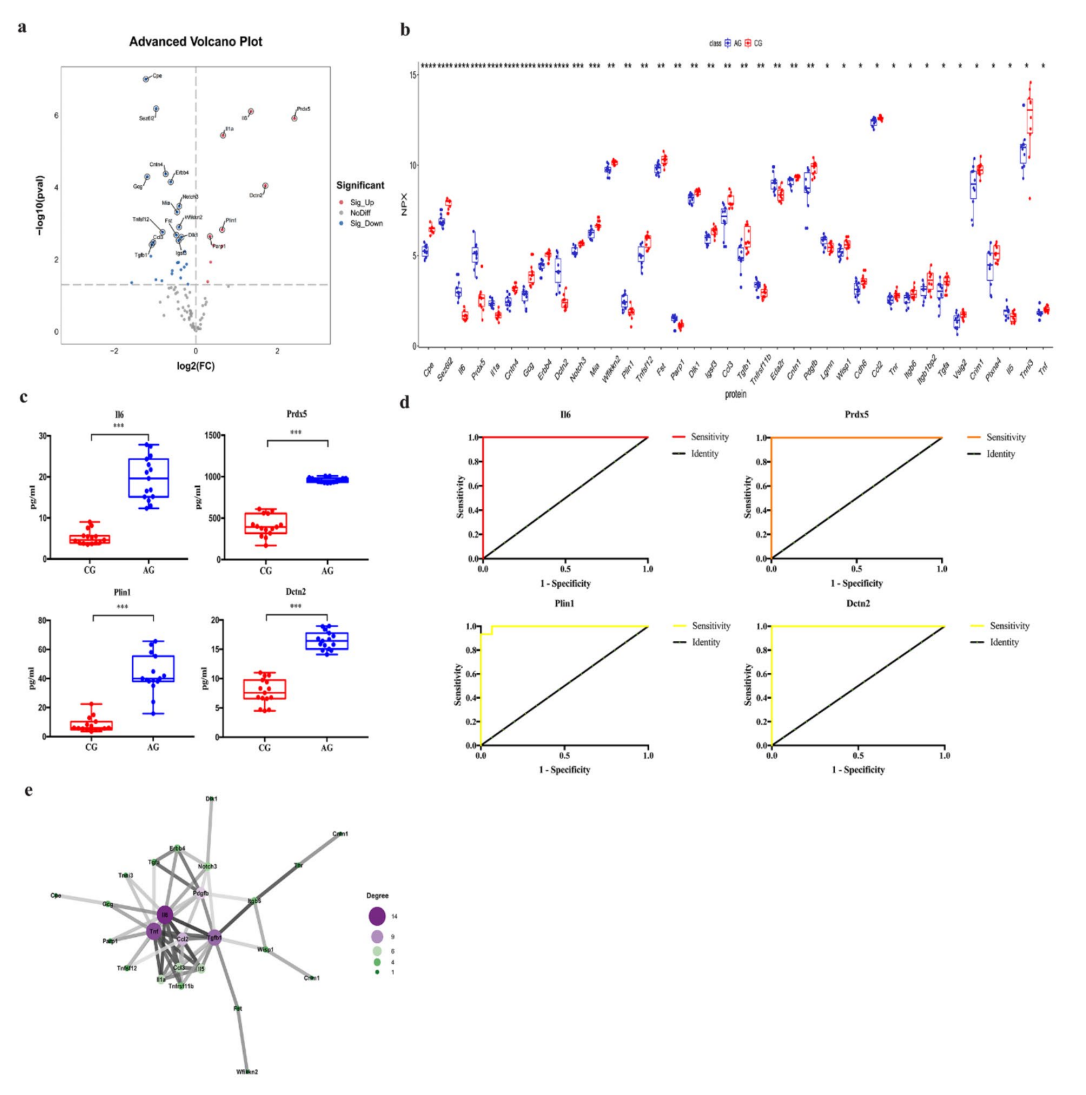
Comparison of the serum inflammation-related proteins between acute compartment syndrome group (AG) and control group (CG). (**a**) Volcano plots showed significant differences of 8 cytokines between AG and CG. Proteins highly expressed in AG were labeled in red. Proteins highly expressed in CG were labeled in blue. Differences between AG and CG were expressed as Log2 (fold change) of plasma on the x-axis and the (-Log10) p value on the y-axis; (**b**) Box plot representing differentially expressed proteins between AG and CG; (**c**)The levels of Il6, Prdx5, Plin1 and Dcnt2 between AG and CG were shown in box plot by ELISA; (**d**) Receiver operating characteristic curve analysis of Il6, Prdx5, Plin1 and Dcnt2 between AG and CG; (**e**) Correlation in protein abundance PPI using String. (CG: control group; AG: acute compartment syndrome group; * <0.05;**<0.01: ***<0.001)

As shown in Tables 1 and 20 differentially expressed inflammation-related proteins were found in two groups, including 14 down-regulated DEPs and 6 up-regulated DEPs (Il6, Il1a, Prdx5, Dctn2, Plin1, and Parp1) (Figs. 2c and 5a–b). ELISA showed that the expression levels of Il6, Prdx5, Dctn2, and Plin1 were remarkably higher in the AG than in the CG (all *p* < 0.001, Fig. 5c). ROC curve analysis was conducted on Il6 [*p* < 0.0001, AUC = 1.000, 95% CI (1.000 − 1.000)], Prdx5 [*p* < 0.0001, AUC = 1.000, 95% CI (1.000 − 1.000)], Dctn2 [*p* < 0.0001, AUC = 1.000, 95% CI (1.000 − 1.000)], and Plin1 [*p* < 0.0001, AUC = 0.996, 95% CI (0.981 − 1.000)] (Fig. 5d; Table 2). The optimal cut-off values were 10.6783 pg/ml, 766.5879 pg/ml, 12.5627 pg/ml, and 14.3623 pg/ml, respectively (Table 2). We also found that Il6 + Prdx5 + Dctn2 + Plin1 [*p* < 0.0001, AUC = 1.000, 95% CI (1.000, 1.000)] had the highest diagnostic accuracy (Fig. 5d; Table 2). Additionally, the PPI network showed that Il6 and Tnf may play an important role (Fig. 5e).

**Fig. 6:**
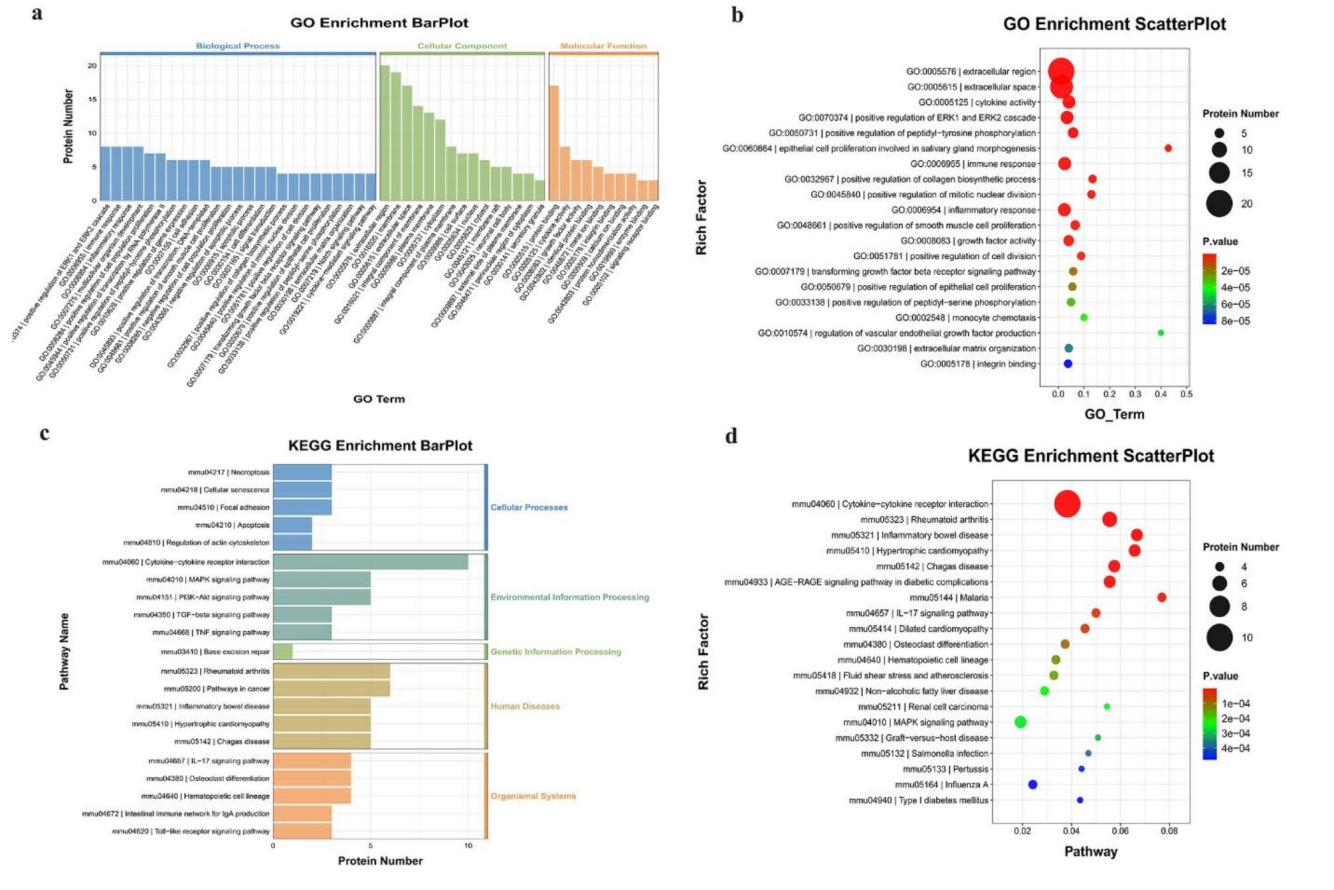
Gene Ontology (GO) terms and Kyoto Encyclopedia of Genes and Genomes (KEGG) of the significant inflammation proteins between AG and CG. (**a**&**b**) GO terms of the significant inflammation proteins between AG and CG; (**c**&**d**) KEEG enrichment analysis of the significant inflammation proteins between AG and CG. (CG: control group; AG: acute compartment syndrome group)

The GO analysis showed enrichment of cytokine activity, the immune response, and the inflammatory response (Fig. 6a–b). KEGG enrichment analysis indicated that cytokine-cytokine receptor interaction, the PI3K-Akt signaling pathway, and the MAPK signaling pathway were enriched (Fig. 6c–d).

### AG vs. FG

**Fig. 7:**
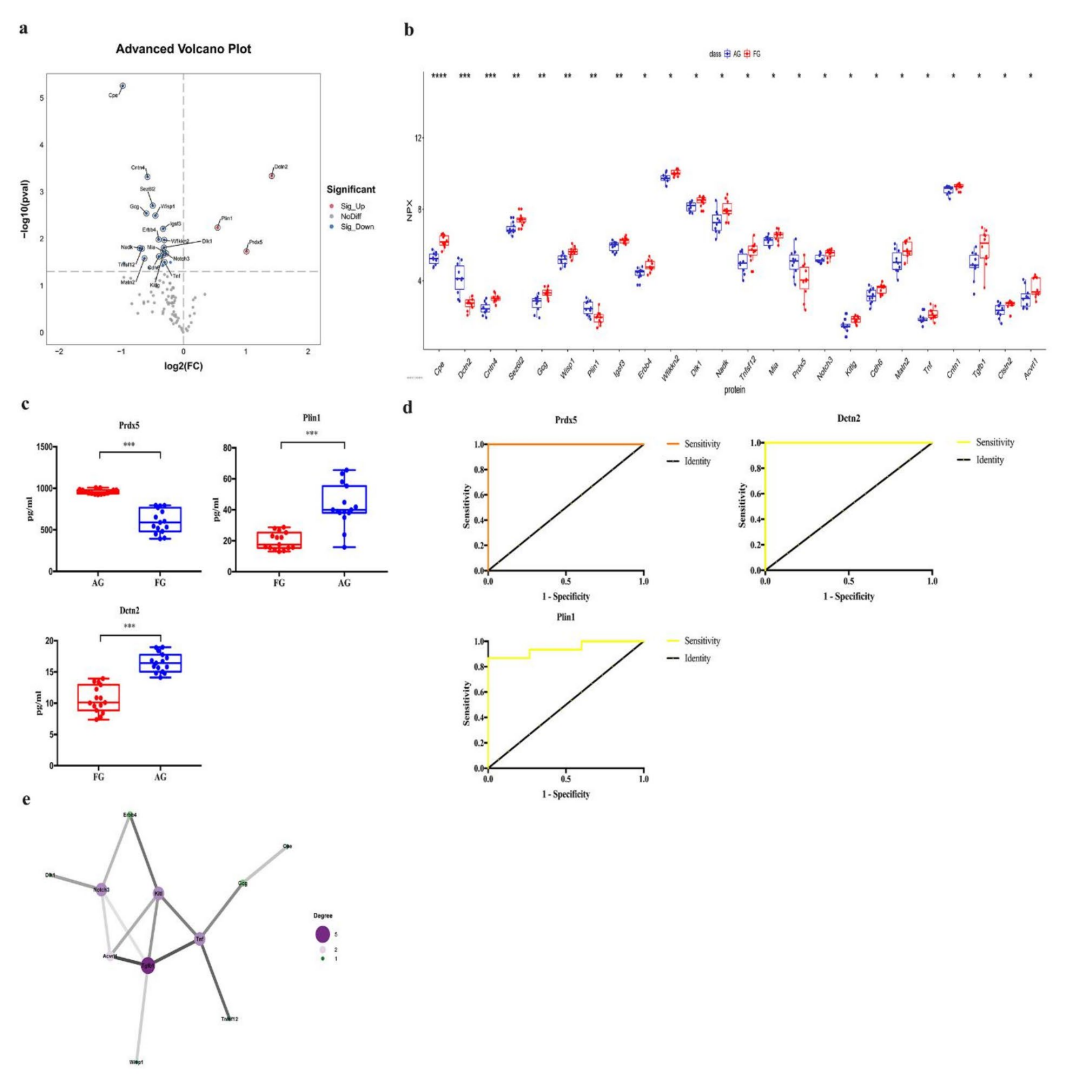
Comparison of the serum inflammation-related proteins between acute compartment syndrome group (AG) and tibial fracture group (FG). (**a**) Volcano plots showed significant differences of 8 cytokines between AG and FG. Proteins highly expressed in AG were labeled in red. Proteins highly expressed in FG were labeled in blue. Differences between AG and FG were expressed as Log2 (fold change) of plasma on the x-axis and the (-Log10) p value on the y-axis; (**b**) Box plot representing differentially expressed proteins between AG and FG; (**c**) The levels of Prdx5, Plin1 and Dcnt2 between AG and FG were shown in box plot by ELISA; (**d**) Receiver operating characteristic curve analysis of Prdx5, Plin1 and Dcnt2 between AG and FG; (**e**). Correlation in protein abundance PPI using String. (AG: acute compartment syndrome group; FG: fracture group; * <0.05;**<0.01: ***<0.001)

As shown in Table 1, eight differentially expressed inflammation-related proteins were found in two groups, including 17 down-regulated DEPs and 3 up-regulated DEPs (Prdx5, Dctn2, and Plin1) (Figs. 2d and 7a–b). ELISA showed that the expression levels of Prdx5, Dctn2, and Plin1 were significantly higher in the AG than in the FG (*p* < 0.001, Fig. 7c). ROC curve analysis was conducted to determine the AUC of Prdx5 [*p* < 0.0001, AUC = 1.000, 95% CI (1.000, 1.000)], Dctn2 [*p* < 0.0001, AUC = 1.000, 95% CI (1.000, 1.000)], and Plin1 [*p* < 0.0001, AUC = 0.942, 95% CI (0.855, 1.000)] (Fig. 7d; Table 2). Their optimal cut-off values were 858.8764 pg/ml, 14.023 pg/ml, and 31.8730 pg/ml, respectively (Table 2). We found that Prdx5 + Dctn2 + Plin1 [*p* < 0.0001, AUC = 1.000, 95% CI (1.000, 1.000)] had the highest diagnostic accuracy (Fig. 7d; Table 2). Additionally, the PPI network showed that Il6 may play an important role in the FG (Fig. 7e).

**Fig. 8:**
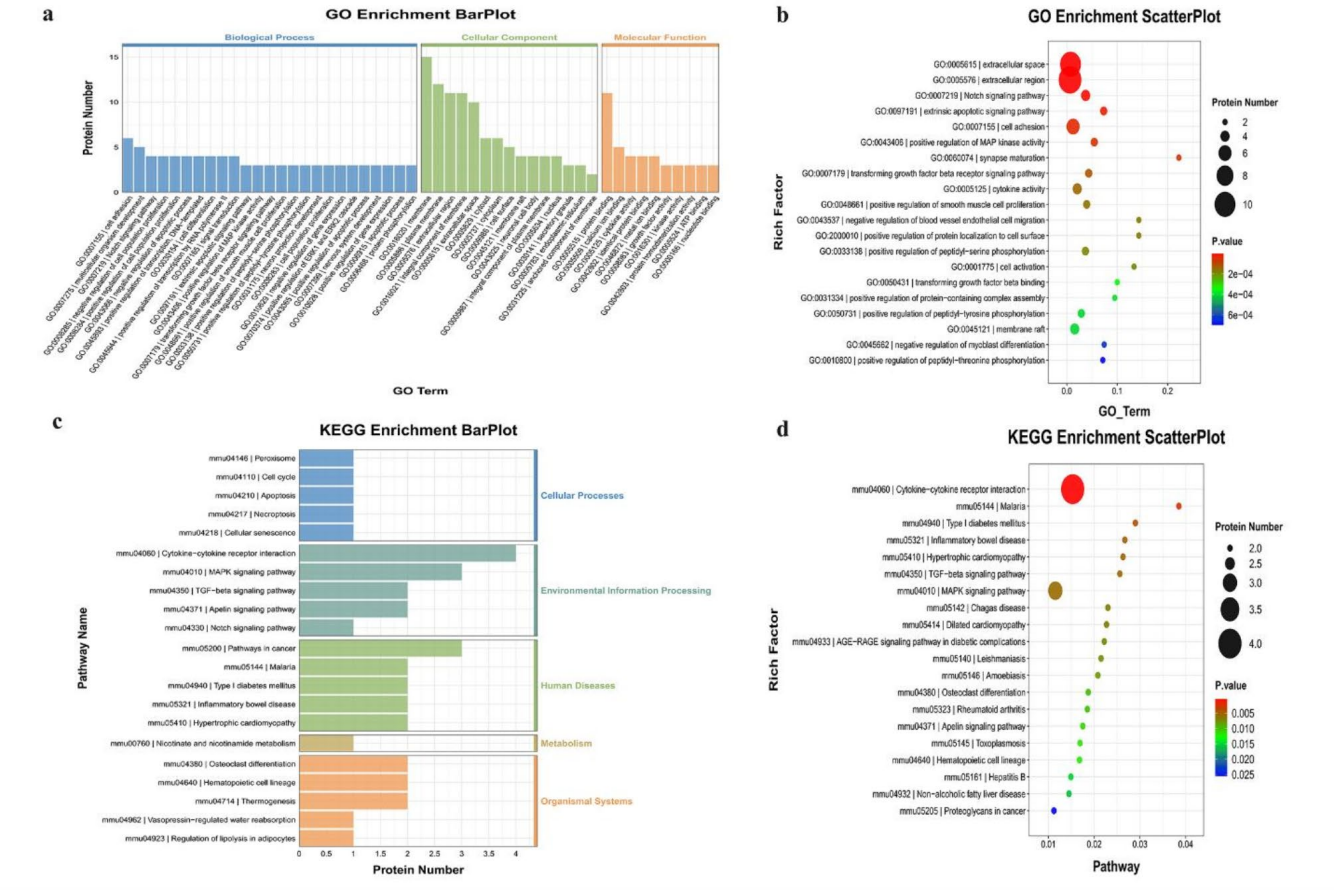
Gene Ontology (GO) terms and Kyoto Encyclopedia of Genes and Genomes (KEGG) of the significant inflammation proteins between AG and FG. (**a**&**b**) GO terms of the significant inflammation proteins between AG and FG; (**c**&**d**) KEEG enrichment analysis of the significant inflammation proteins between AG and FG. (CG: control group; FG: fracture group)

In the GO enrichment analysis, the Notch signaling pathway was enriched (Fig. 8a–b). KEGG enrichment analysis indicated that cytokine-cytokine receptor interaction, the MAPK signaling pathway, and the TGF-β signaling pathway were enriched (Fig. 8c–d).

## Discussion

Despite substantial improvement in its management, there is a sustainable need for reliable and rapid methods to diagnose ACS, a serious complication of tibial fractures, and to improve the therapeutic efficacy, which may influence patient outcomes. The need for a fast interventional step is especially important because ACS may cause muscle necrosis, function deficits, or amputation^1,2,7^. Notably, there is no available biomarker of ACS with high specificity, which presents an enormous challenge for orthopedic surgeons to diagnose this condition without delay. Our previous study using complete blood counts showed that Neutrophils and creatine kinase isoenzymes levels were related to ACS, but limited inflammation-related indicators were observed. Additionally, our recent studies found variations of inflammatory cytokines in patients with fracture blister fluid and plasma using Olink Proteomics^15^. Therefore, we used the inflammatory panel of Olink Proteomics to identify biomarkers of ACS in the current study.

Our findings showed that the levels of Il6 and Prdx5 in the FG and Il6, Prdx5, Dctn2, and Plin1 in the AG were significantly higher than those in the CG. Notably, compared with the FG, Prdx5, Dctn2, and Plin1 exhibited higher expression in the AG. Additionally, we identified 58.8764 pg/ml, 14.023 pg/ml, and 31.8730 pg/ml as the cut-off values of Prdx5, Dctn2, and Plin1 to predict ACS in rats. Similarly, the cut-off values of Il6, Prdx5, Dctn2, and Plin1 to distinguish ACS rats from healthy rats were 10.6783 pg/ml, 766.5879 pg/ml, 12.5627 pg/ml, and 14.3623 pg/ml, respectively. Furthermore, the combination of these proteins had the highest diagnostic accuracy.

In all types of cells, reactive oxygen species (ROS) are usually generated and removed and have physiological and pathological effects^16^. Prokaryotes and eukaryotes both have high levels of Prdx5 protein, which is a member of the antioxidant protein superfamily. There are six members in the Prdx family in nursing animals: Prdx1–Prdx6. Prdx5, which is mostly found in the cytoplasm, mitochondria, peroxisomes, and nucleus, was initially described in 1992^17^. According to some studies, Prdx proteins have the ability to scavenge free radicals and act as potent antioxidants. They can also decrease peroxide and superoxide levels via thioredoxin and can be activated by the ROS/JNK pathway during oxidative stress^18,19^. Researchers also discovered that the expression of Prdx5 is connected to cell division, proliferation, and signal transduction in their functional analysis of the protein^20–22^. Substantial evidence indicates that Prdx5 plays an important role in various diseases, including liver disease and non-small-cell lung cancer^23,24^. However, the relationship between Prdx5 and ACS in rats remains unclear. We found that the level of Prdx5 was highest in the AG and lowest in the CG, indicating that injury-induced Prdx5 stimulation contributed to the antioxidant effect. Furthermore, we identified 858.8764 pg/ml as the cut-off value of Prdx5 to predict ACS in rats with fracture and 766.5879 pg/ml as the cut-off value of Prdx5 to predict ACS in healthy rats. These results indicate that the serum level of Prdx5 is a potential marker that can differentiate ACS from fracture.

Il-6 is an important immunomodulatory cytokine that is involved in numerous disease processes, including autoimmune diseases and chronic inflammatory conditions^25^, and it is considered a diagnostic and prognostic tool and a potential therapeutic target for various injuries, such as traumatic brain injury, post-infarction cardiac injury, and acute kidney injury^26–28^. To our knowledge, no study has focused on the relationship between Il-6 and ACS in a rat model. We found that the levels of Il-6 in the AG and FG were higher than that in the CG, indicating that rats with fracture and those with ACS have greater inflammatory responses than healthy rats. Interestingly, there was no significant difference in the inflammatory response between the FG and AG, which implied that the inflammatory response levels of both groups were similar. Furthermore, we identified 10.6783 pg/ml as the cut-off value of Il6 to predict ACS in healthy rats. These results indicate that the serum level of Il6 is a potential marker to differentiate ACS rats from healthy rats.

Because it regulates the access of lipases to neutral lipids in lipid droplets, Plin1 is essential for maintaining lipid homeostasis. PLIN1 either restricts lipase access to stored triglycerides or promotes hormonally induced lipolysis, depending on the energy status of the organism^29^. Numerous in vivo and in vitro investigations, including those involving Plin-null mice, have verified that elevated basal lipolysis is a consequence of either decreased or absent Plin1. Plin1-deficient mice exhibited reduced stimulated lipolysis and increased basal lipolysis. Plin1 also has a substantial correlation with lipid metabolism in chickens, which is consistent with the findings in mammals. Additionally, Plin1 acts as an important protective regulator against adipose tissue inflammation. The relationship between Plin1 and ACS rats is still debated. Our findings showed that the level of Plin1 in the AG was higher than those in the FG and CG, indicating that injury-induced Plin1 stimulation protectively decreases tissue inflammation. Furthermore, we identified 31.8730 pg/ml as the cut-off value of Plin1 to predict ACS in fracture rats and 14.3623 pg/ml as the cut-off value to predict ACS in healthy rats. These results indicate that the serum level of Plin1 is a potential marker to differentiate ACS from fracture.

Dctn is a multiple-subunit protein compound involved in the activation of most forms of cytoplasmic dynein in eukaryotes^31^. Dctn connects to cytoplasmic dynein, dynein cargo adaptors, and microtubules^32^. Six subunits of dynactin, DCTN1, DCTN2, DCTN3, DCTN4, DCTN5, and DCTN6, have been identified, and all subunits are encoded by their corresponding genes^32^. Studies have demonstrated various pharmacological properties of DCTN, including antitumor^33^, anti-inflammatory, and antinociceptive effects^34^. Our findings showed that the level of Dctn2 in the AG was higher than that in the FG and CG, indicating that injury-induced Dctn2 plays a protective role against inflammation. Furthermore, we identified 14.023 pg/ml as the cut-off value of Dctn2 to predict ACS in fracture rats and 12.5627 pg/ml as the cut-off value to predict ACS in healthy rats. These results indicate that the serum level of Dctn2 is a potential marker to differentiate ACS from fractures.

Although, to the best of our knowledge, this is the first study to investigate the potential biomarkers of ACS in rats using Olink Proteomics, some limitations should be noted. First, we only focused on the potential role of inflammation-related indicators in the prediction of ACS and neglected other possible factors such as genetics and environmental factors. In future studies, we will comprehensively consider these factors and carry out a more comprehensive clinical study. Second, we did not observe the dynamic variations of relevant inflammatory markers. Further well-designed prospective studies are needed to verify our results.

In summary, we identified Il6, Prdx5, Dctn2, and Plin1 as potential biomarkers of ACS compared with tibial fractures in rats as well as the significance of the combination of Il6, Prdx5, Dctn2, and Plin1 to predict ACS compared with the healthy condition. Furthermore, we found their cut-off values and the combinations with the highest diagnostic accuracy, providing a new method to rapidly assess the risk of ACS and manage early targeted interventions.

## Data Availability

Please contact the correspondence authors if request on data.
